# Problems in Diagnosis

**Published:** 1921-12

**Authors:** Julio Endelman


### PROBLEMS IN DIAGNOSIS

BY JULIO ENDELMAN, D.D.S.

The purpose of diagnosis is the corelation of radiographic and clinical findings, history and clinical manifestations for the ultimate purpose of determining upon a plan of rational treatment. In dentistry, the plan of treatment that suggests itself frequently is of radical nature, and the problem of replacements enters into the question to such an extent as to greatly complicate the issue. When extractions are indicated, as the only means of eradicating chronic infections, it is of vital importance that plans be formulated at the time to supply the patient with artificial substitutes fulfilling anatomical and physiological requirements. To simply order the extraction of a number of teeth without concern of the future masticatory efficiency of the patient, is decidedly unfair.

A dental diagnosis should begin with a general survey of the patient from the standpoint of his general health. The patient's appearance, the nature of his movements, the color of the skin and mucous membranes will often help in framing a diagnosis. Next in order should invariably be a complete personal and family history, such a history to include data relating to causes of death of members of the patient's family and all information concerning the patient's past and present general health. If the patient should be under the care of a physician, much valuable information can be secured by adopting an attitude of sympathetic co-operation with him. The physician may have laboratory findings frequently of indispensable value in framing a dental diagnosis. Before undertaking the care of patients, the existence of such chronic maladies as diabetes, tuberculosis, syphilis, cardiac insufficiency, rheumatic and arthritic difficulties and endocrine disturbances should be ascertained. The existence of any one or combination of these diseases has an important bearing in our final decisions. Armed with this information and with correctly secured radiograms of all the teeth and surrounding osseous tissues, an examination is now undertaken of each individual tooth, of the investing tissues and of the lining of the mouth. This must include general appearance of the mouth, missing or supernumerary teeth, operative and prosthetic restorations, condition of the investing and periapical structures, root canals, direction of occlusal stress, whether normal or abnormal, the presence of sinuses, neoplasms, etc., etc. The examination in detail should now be made of each individual tooth, with special reference to caries, incipient or advanced, and gingival and peridental infections, noting degree of involvement and looseness of teeth so affected. This examination is conducted with the help of radiograms, and, whenever possible, the physical findings are compared with the evidence presented in the radiograms, and vice versa. With reference to incipient caries, dentists are, as a general rule, neglectful of these danger signals, although nothing in dentistry is pregnant with greater possibilities than the eradication of beginning caries. To pass over incipient caries, because of superficial and hasty examination, is nothing short of a professional crime.

If areas of radiolucency are found surrounding the apices of roots, unless such evidence should be overwhelming, as in the case of roots with partially or completely filled root canals, it is advisable to subject the pulps to a vitality test, as the radiogram is unreliable as an indicator of pulp conditions; the best that may be said for it in this connection is that by comparison with the appearance of the pulps of other teeth in the same mouth some suggestive information may be obtained. An infection in the periapical tissues may exist in the presence of a pulp not totally devitalized and further artifacts in radiograms may simulate the radiolucency which results from chronic osteomyelitis and lead, in the case of superficial examinations, to the useless sacrifice of teeth.

While we consider that properly taken radiograms are indispensable in oral and dental diagnosis, we deplore the tendency to determine the condition of the mouth and teeth upon that basis only. Diagnosis must be based upon the etiology and pathology of disease and to this should be added an extensive clinical experience.

Teeth that call for extraction are, as a general rule, affected with either periapical infections or pyorrheal involvement. No fast rule can be laid down as to when teeth affected with either or both of these conditions should be extracted. It is not the extent alone of the periapical infection that should be the deciding factor, for a small lesion may be the source of as much injury, metastatically, as a large one. Other factors should be seriously considered, such as the patient's general health, the vital resistance, whether normal or sub-normal, the patient's age, the position of the tooth, its physiologic and cosmetic roles and its anatomical peculiarities. This rule may be safely laid down. In the presence of systemic involvements, there is no possible justification in retaining teeth with chronic periapical infections. The successful treatment of root canals is not any more of a certainty than the treatment of any other deep-seated infection, and even when successful, months must elapse before any indication may be had that the eradication of the infection will be accomplished. Subjecting patients to the stress of such an infection may terminate fatally. As much harm may result from one or more infected teeth acting in the capacity of depressants of body defences, as if such teeth were actually the source of systemic lesions in remote areas. The fact that under certain conditions the extraction of teeth is recommended should not be interpreted as a disbelief in the possibility of the recovery of teeth the seat of chronic periapical infections. If the patient's resistance is normal, or nearly so, and the area of involvement is not too extensive, and, further, if the root-canal is rendered sterile, there is no biological or pathological reason why regeneration of the rarefied area should not occur and, indeed under these favorable conditions it does actually occur.—*Pacific Gazette.*

